# A differential network analysis approach for lineage specifier prediction in stem cell subpopulations

**DOI:** 10.1038/npjsba.2015.12

**Published:** 2015-11-12

**Authors:** Satoshi Okawa, Vladimir Espinosa Angarica, Ihor Lemischka, Kateri Moore, Antonio del Sol

**Affiliations:** 1 Luxembourg Centre for Systems Biomedicine (LCSB), University of Luxembourg, Esch-sur-Alzette, Luxembourg; 2 Ichan School of Medicine at Mount Sinai, New York, NY, USA

## Abstract

**Background:**

Stem cell differentiation is a complex biological process. Cellular heterogeneity, such as the co-existence of different cell subpopulations within a population, partly hampers our understanding of this process. The modern single-cell gene expression technologies, such as single-cell RT-PCR and RNA-seq, have enabled us to elucidate such heterogeneous cell subpopulations. However, the identification of a transcriptional regulatory network (TRN) for each cell subpopulation within a population and genes determining specific cell fates (lineage specifiers) remains a challenge due to the slower development of appropriate computational and experimental workflows. Here, we propose a computational differential network analysis approach for predicting lineage specifiers in binary-fate differentiation events.

**Methods:**

The proposed method is based on a model that considers each stem cell subpopulation being in a stable state maintained by its specific TRN stability core, and cell differentiation involves changes in these stability cores between parental and daughter cell subpopulations. The method first reconstructs topologically different cell-subpopulation specific TRNs from single-cell gene expression data, literature knowledge and transcription factor (TF)–DNA binding-site prediction. Then, it systematically predicts lineage specifiers by identifying genes in the TRN stability cores in both parental and daughter cell subpopulations.

**Results:**

Application of this method to different stem cell differentiation systems was able to predict known and putative novel lineage specifiers. These examples include the differentiation of inner cell mass into either primitive endoderm or epiblast, different progenitor cells in the hematopoietic system, and the lung alveolar bipotential progenitor into either alveolar type 1 or alveolar type 2.

**Conclusions:**

The method is generally applicable to any binary-fate differentiation system, for which single-cell gene expression data are available. Therefore, it should aid in understanding stem cell lineage specification, and in the development of experimental strategies for regenerative medicine.

## Introduction

Stem cell differentiation is a complex biological process.^1,2^ Our understanding of this process is partially hampered by the heterogeneity of stem cell populations. Indeed, different cell subpopulations co-existing within a population have different propensities for cell fate decision determined by their specific transcriptional regulatory networks (TRNs)s. Hence, conventional bulk gene expression profiling and ChIP-seq approaches appear to be suboptimal for studying cell differentiation.^3^ Modern single-cell gene expression technologies, such as single-cell RT-PCR and RNA-seq, have enabled us to elucidate heterogeneity in different stem cell systems.^3–8^ However, the development of general computational methods for systematically predicting lineage specifiers within heterogeneous cell populations has been lagging behind. Here we introduce a general method for predicting lineage specifiers in binary-fate differentiation events based on the reconstruction and analysis of cell subpopulation-specific TRNs.

Our method is based on a model that considers stem cell subpopulations to be in a stable state, and attempts to identify cell subpopulation-specific network cores conferring such stability. The model assumes that cell differentiation involves changes in such stability cores between parental and daughter cell subpopulations and the prediction of lineage specifiers is carried out by identifying genes that reside in the stability cores in both parental and daughter cell subpopulations. Our model further presumes that lineage specifiers for one daughter cell subpopulation should be differentially active in comparison with the other daughter cell subpopulation. By considering two mutually exclusive lineages, the method is able to exclude general prodifferentiation genes that are active in both lineages in comparison with the parental cell subpopulation. We particularly focused on a directed network motif, the strongly connected component (SCC), in which any network node is reachable from any other node. As an SCC is a cluster of feedback loops (circuits), it can autonomously sustain stable steady states without external stimuli. For this reason, we assumed that SCCs confer stability to cellular phenotypes and perturbation of transcription factors (TFs) in SCCs could lead to destabilization of phenotypes. Indeed, this notion has previously been used for predicting cell fate determinants.^9,10^ In order to build subpopulation-specific TRNs, we first combined different sources of information, including single-cell gene expression data, literature knowledge, and TF–DNA binding site prediction. We then employed a modified version of the network-pruning algorithm previously developed by us to identify cell subpopulation-specific regulatory interactions.^11^

The method was applied to three different binary-fate stem cell differentiation systems for which high-quality single-cell gene expression data are available. These examples include the differentiation of inner cell mass (ICM) into either primitive endoderm (PE) or epiblast (EPI),^4^ the differentiation of different progenitor cells in the hematopoietic system (hematopoietic stem cell (HSC) into either multipotent progenitor (MPP) or megakaryocyte–erythroid progenitor (MEP), MPP into common myeloid progenitor (CMP) or common lymphoid progenitor (CLP), and CMP into either MEP or granulocyte–macrophage progenitor (GMP)),^6^ and the differentiation of lung alveolar bipotential progenitor (BP) into either alveolar type 1 (AT1) or alveolar type 2 (AT2).^8^ The method predicted *Gata6* for PE and *Klf2* and *Nanog* for EPI, which is in full agreement with previous experimental evidence.^12–15^ In addition, well-known lineage specifiers in the hematopoietic system, such as *Cebpa*,^16^ *Gata1*,^17^ *Gfi1*^18^ and *Spi1* (PU.1)^19^ were correctly predicted for appropriate subpopulations. Finally, the application to the relatively understudied lung BP developmental system predicted candidate lineage specifiers with prior associations with lung development, including *Hes1*^20^ and *Srf*.^21^

To our knowledge, this is the first computational method that, without prior knowledge of potential candidate genes, systematically predicts cell lineage specifiers in stem cell subpopulations based on the reconstruction and analysis of their specific TRNs derived from single-cell gene expression data. Indeed, the importance of deriving subpopulation-specific TRNs from single-cell gene expression data here recently been discussed in.^22^ The authors showed that TRN models generated from population-bulk expression data do not account for functional relationships between genes in each cell subpopulation. Furthermore, it has been previously shown that cell subpopulation-specific TRNs showed significant rewiring during differentiation.^3^ Hence, TRNs that are differentially reconstructed for different cell subpopulations appear to provide a biologically more realistic scenario than the reconstruction of a single TRN representing multiple cell subpopulations. The method is generally applicable to any binary-fate differentiation system, for which single-cell gene expression data are available. Therefore, it should aid in understanding stem cell lineage specification, and in the development of experimental strategies for regenerative medicine.^1^

## Materials and Methods

### Stem cell differentiation model

In this model stem cell subpopulations can be regarded as stable steady states (i.e., attractors) in the gene expression landscape determined by their TRNs. We assume that if a system is closed and does not contain a feedback loop (circuit), then the gene expression states in the system will eventually become all 0, since keeping a gene in the active state requires constant input from other (or its own) gene products. As SCCs consist of a cluster of circuits, they can autonomously sustain stable steady states without external stimuli. For this reason, we further assume that SCCs confer stability to cellular phenotypes and perturbation of TFs in SCCs could lead to destabilization of phenotypes. This notion has previously been used for predicting cell fate determinants.^9,10^ Since differentiation signals need to destabilize the attractor state of the parental subpopulation and to stabilize that of the daughter subpopulation, we propose that genes involved in lineage specification belong to SCCs in both parental and daughter cell subpopulations. Furthermore, lineage specifiers for one daughter cell subpopulation should be differentially active in comparison with the other daughter cell subpopulation.

### Single-cell gene expression data processing

The single-cell gene expression data sets for mouse ICM differentiation,^4^ HSC differentiation^6^ and lung BP differentiation^8^ were obtained from Gene Expression Omnibus. Transcription factors/regulators (TFs) annotated at (http://www.bioguo.org/AnimalTFDB/)^23^ were extracted from these data sets, resulting in 26, 55 and 900 total TFs, respectively. In the first two RT-PCR data sets the normalized *C*_T_ values were converted into gene expression values by applying a base 2 exponential transformation as described in.^24^ For the third data set, the FPKM values were used and the missing values were imputed with the lowest expression value. We used the same single-cell sample classes as in the respective data sets. The ICM, PE and EPI subpopulations were previously unbiasedly classified by principle component analysis (PCA),^4^ the HSC, MPP, CMP, MEP, GMP and CLP subpopulations were classified by combinations of surface markers,^6^ and the BP, AT1 and AT2 subpopulations were previously classified by PCA.^8^

### Gene expression Booleanization based on cell population heterogeneity

For Booleanization of the gene expression data, we compared the significance of the expression of each gene in each subpopulation against the background distribution formed by the union of the expression values of all cell subpopulations that co-exist at a given moment. For example, the ICM and trophoectoderm (TE) cell subpopulations co-exist in the 32-cell stage cells and therefore, the expression of ICM genes was compared against the background expression formed by both ICM and TE cells. Similarly, the Booleanization of the gene expression of PE and EPI was performed against the background expression formed by all 64-cell stage cells (i.e., PE, EPI and TE (64C)). The six subpopulations of the HSC data set co-exist in the mouse bone marrow; therefore, the background expression was formed by combining all the six subpopulations. The BP, AT1 and AT2 cell subpopulations also co-exist at embryonic day 18.5 and the background expression was formed by combining all these three subpopulations. As the gene expression values did not follow a normal distribution, the significance *P*-value of a gene against the background expression was non-parametrically computed using the one-sided Mann–Whitney–Wilcoxon test.^25^ The cutoff of *P*-value ⩽ 0.4 was set, below which the expression of a gene was considered differentially active ‘1’, and otherwise ‘0’ (i.e., not significantly differentially active) in a Boolean manner. The Booleanized expression data are available in Supplementary Tables S1–S3.

### TRN reconstruction

To increase the confidence of TRN interactions, we combined three different source of information.

1. Network inference from literature knowledge: The information about experimentally validated interactions among TFs was retrieved from the MetaCore server.^26^ The interaction types ‘Transcriptional regulation’, ‘Binding’ and ‘Influence on expression’ were selected. These data include the information on the directionality of the interactions and its mode of action (i.e. activation or inhibition, or unspecified otherwise). This set of interactions included distal element-mediated interactions.
2. Network inference by TF–DNA binding-site prediction: The prediction of TF–DNA binding site was carried out using the MATCH tool.^27^ The information regarding the transcription start sites (TSSs) was obtained from the RefSeq database.^28^ Promoter sequences comprising 2,000 base pairs upstream and 1,000 base pairs downstream from TSSs were obtained using twobitToFa utility and 0.2 bit genome sequence files (hg19, mm10) from UCSC (http://hgdownload.soe.ucsc.edu/downloads.html).
3. Network inference from single-cell gene expression data: Single-cell gene expression data allow us to infer more realistic co-expression relationships between genes, which can significantly increase the reliability of network inference.^29^ As the gene expression patterns between gene pairs were not following a normal distribution, mutual information was used as a statistical metric since it makes no assumption about the underlying statistical distribution. For this purpose, we used MRNET^30^ implemented in R,^31^ which employs the maximum relevance−minimum redundancy. Next, we filtered out weakly inferred interactions by computing the null distribution of interaction strengths. To do this, a randomized expression matrix of a given cell subpopulation was made by randomly shuffling the gene expression values of each single-cell sample and the same MRNET inference was performed on this randomized expression matrix for inferring the interactions corresponding to the null distribution. This procedure was repeated 10,000 times and a significance *P*-value for each interaction was computed against the interaction strength inferred from these randomized expression matrices. The *P*-values were then ranked and the interactions among the top half of the total interactions were considered as putative interactions.

These three different sources of interactions were combined to reconstruct raw TRNs. We took the union of the intersection between 1 and 3, and the intersection between 2 and 3 (Supplementary Figure S1). The rationale behind this approach is that: (i) the interactions in 1 and 2 are not cell-type specific and mainly come from bulk data that may contain a heterogeneous population of cells, therefore 3 can add cell-subpopulation specificity to each TRN; and (ii) the interactions in 1 are already reported interactions, therefore, adding the intersection between 2 and 3 could add novel, proximal element (promoter)-mediated transcriptional interactions that were supported by two different information sources. Although we could not add novel interactions mediated by distal elements, to our knowledge, no comprehensive approach for accurately linking distal elements with regulated genes is available.

Raw TRNs (Supplementary Information 23–34) were then contextualized (i.e., pruned) to the Booleanized gene expression profiles of each cell subpopulation (Supplementary Information 35–46) using a newly implemented, improved version of the method previously developed in our group,^11^ (Supplementary Information 1–4). This algorithm assumes that each cellular phenotype is a stable steady-state attractor of a Boolean network, and removes edges that are inconsistent with the Booleanized gene expression data. The Boolean simulation was conducted using the pbn-matlab-toolbox (http://code.google.com/p/pbn-matlab-toolbox/downloads/list) with a synchronous updating scheme. This tool box defines the logic rule for genes receiving multiple interactions, such that the edge strengths of activating and inhibiting edges acting on a gene are compared and the stronger one dominates (i.e., the threshold rule). If the edge strengths of activations and inhibitions are equal, then the state of the target gene was set to remain in its current state. All edge weights were set to 1. On purpose, specific interaction logic rules were not incorporated into our workflow in order to keep the method applicable to systems even without any prior knowledge of logic rules. Recently, a few attempts have been made to derive more customized Boolean logic rules using single-cell gene expression data.^32,33^ The former used a TRN derived from population bulk data as a reference topology for inferring Boolean logic rules, whereas the latter derived a TRN topology and its logic rules solely from single-cell gene expression data. In the current study, however, we assumed and derived different TRN topologies among different cell subpopulations from single-cell gene expression data in combination with other biological information sources. Therefore, these approaches were not suitable for our case.

During network pruning, ‘unassigned’ interactions (i.e., interactions for which the effect of activation or inhibition is unknown) were randomly assigned ‘activation’ or ‘inhibition’ in the first generation of the GA. Then, the algorithm computed the attractor state of that network, calculated the mismatch with the target expression state and the best solutions in this generation of the GA were selected and used for the next generation. In this way, it shifted the probability for assigning ‘activation’ or ‘inhibition’ to better solutions over generations. The optimization function was designed to minimize mismatches between the simulated Boolean attractor and gene expression data. When a gene has >30 incoming edges, the number was reduced to 29 by randomly removing the incoming edges to reduce the computational load. For the BP system, the contextualization was performed on the SCC of the entire raw TRN, as the genetic algorithm could not converge when the entire TRN was used due to its size. The contextualized network was visualized in Cytoscape (version 2.7.0).^34^

### Lineage specifier prediction

Our model of stem differentiation assumes that lineage specifiers reside in autonomous network stability cores, namely SCCs. However, SCCs consist of smaller SCCs with different regulatory influences on the entire network. For this reason, we previously performed hierarchicalization of SCCs to find the SCCs that reside at the top of this hierarchy by transforming networks into directed acyclic graphs.^9^ However, this transformation requires some topological changes. In addition, we observed that networks with a high number of genes, such hierarchicalization resulted in many SCCs at the same hierarchical level.

Therefore, in order to identify SCCs that have a high regulatory influence on the entire network, we first decomposed SCCs into smaller SCCs and the number of incoming edges (i.e., in-degree interface) was subtracted from the number of out-going edges (i.e., out-degree interface) for each of these SCCs. Only active genes (i.e., those having ‘1’ in Booleanized expression) were used for this calculation, since not differentially active genes, by definition, have the basal level influence on the TRN and differentially active genes have the major, cell subpopulation-specific influence on the TRN. The decomposition of SCCs was performed by first identifying elementary circuits formed by shortest paths from each node to itself using the Yen’s algorithm^35^ implemented in MATLAB (http://www.mathworks.com/matlabcentral/fileexchange/32513-k-shortest-path-yen-s-algorithm). For these circuits, SCCs were formed by extracting the network (interactions) among the constituent genes of the circuit. The attractor was computed for each of these SCCs using the gene expression data as the starting state. SCCs whose attractor states were not 100% identical to the attractor of the entire contextualized network, and/or to their starting gene expression data were discarded. Then, the SCCs were ranked by the degree difference (described above), and those that were ranked among the top were chosen as the ‘most influential SCCs’. To consider the difference in the number of TFs in the networks between the used RT-PCR and RNA-seq data (20–50 TFs and 200–250 TFs, respectively), the top five and top ten most influential SCCs were taken, respectively. Finally, TFs that were ‘1’ in one daughter cell subpopulation but ‘0’ in the other daughter cell subpopulation with more than 2-fold change in the expression value were selected (Supplementary Information 47–56). If these selected TFs were present in the most influential SCCs of both daughter cell subpopulation (where the TFs are active) and parental cell subpopulation, then they were considered candidate lineage specifiers. The programme codes for most influential SCC finding and lineage specifier prediction are available in Supplementary Information 5–10.

## Results

**Figure 1:**
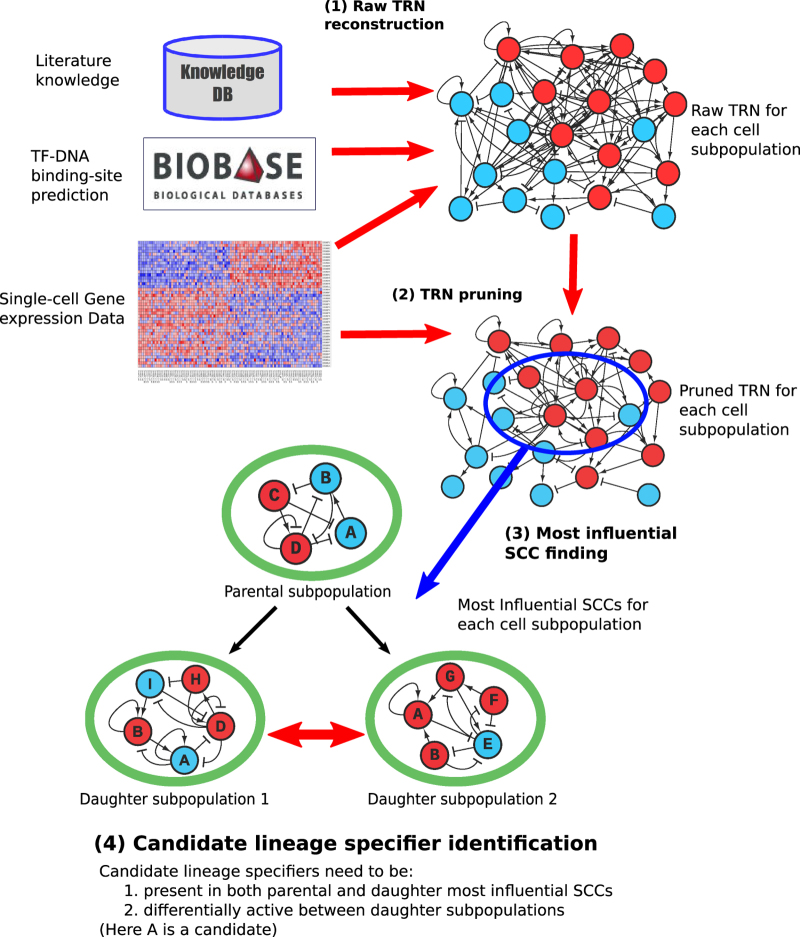
Schematic view of the proposed method for predicting lineage specifiers of stem cell differentiation using single-cell gene expression data. Candidate lineage specifiers are identified in four steps. First, a raw TRN is reconstructed using single-cell gene expression data, literature knowledge and TF–DNA binding-site prediction. This TRN is then contextualized by removing edges that are inconsistent with Booleanized gene expression data. Then, the most influential SCCs are identified in the TRN (see Materials and Methods). In parallel, TFs that are over-expressed in one daughter subpopulation in comparison to the other daughter cell subpopulation are identified. These differentially active TFs that are also present in the most influential SCCs of both parental and daughter cell subpopulations are considered candidate lineage specifiers.SCC, strongly connected component; TF, transcription factor; TRN, transcriptional regulatory network.

The overview of the proposed method is shown in Figure 1 and the details are described in the Materials and Methods section. First, a raw TRN for each cell subpopulation was reconstructed by combining literature-based interactions, predicted TF–DNA binding interactions and single-cell gene expression-based network inference (Supplementary Figure S1). We then performed network contextualization of the TRN by removing interactions that are inconsistent with Booleanized gene expression states. Finally, candidate lineage specifiers were identified as TFs that were differentially active in one daughter cell subpopulation in comparison to the other daughter subpopulation, and were present in the most influential SCCs in both parental and daughter cell subpopulations. Although this current approach does not predict a combination of lineage specifiers that need to be perturbed at the same time, to our knowledge, in most of the known differentiation cases perturbation of one single TF is enough to trigger the transition. Therefore, we expect that our approach is sufficient for most differentiation events.

### Prediction of lineage specifiers in different systems

**Figure 2:**
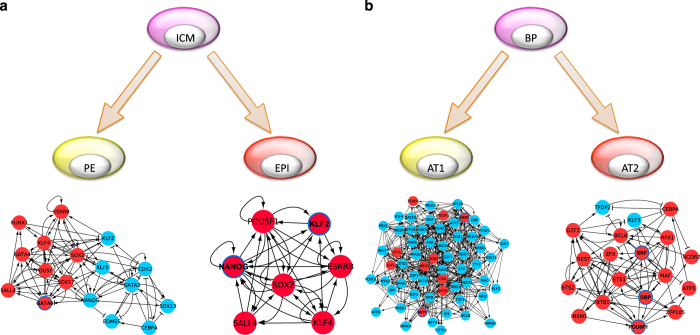
Most influential SCCs of TRNs for binary bifurcations during early embryonic development and lung BP development. (**a**) Differentiation of ICM into either to PE or EPI. (**b**) Differentiation of BP into either AT1 or AT2. Red and blue nodes indicate not over-expressed and over-expressed genes, respectively. Pointed arrows indicate activation and blunted arrows indicate inhibition. Genes with a colored surrounding circle with bold-font name represent predicted lineage specifiers. EPI, epiblast; ICM, inner cell mass; PE, primitive endoderm; SCC, strongly connected component; TRN, transcriptional regulatory network.

**Figure 3:**
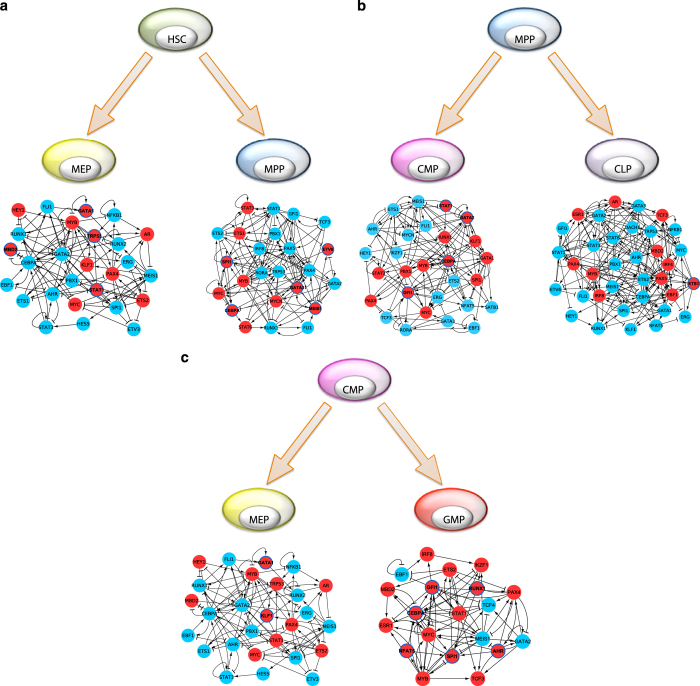
Most influential SCCs of TRNs for three binary bifurcations during hematopoiesis. (**a**) Differentiation of HSC into either MEP or MPP. (**b**) Differentiation of MPP into either CMP to CLP. (**c**) Differentiation of CMP into either MEP and GMP. The graphical properties are described in Figure 2. CLP, common lymphoid progenitor; CMP, common myeloid progenitor; GMP, granulocyte–macrophage progenitor; MPP, multipotent progenitor; MEP, megakaryocyte–erythroid progenitor; SCC, strongly connected component; TRN, transcriptional regulatory network.

The method was applied to three different binary-fate stem cell differentiation systems for which high-quality single-cell gene expression data are available. The first example^4^ is the differentiation of ICM into either PE or EPI (Figure 2a). The second example is the hematopoietic system,^6^ in which HSC differentiates into either MPP or MEP, MPP differentiates into CMP or CLP, and CMP differentiates into either MEP or GMP (Figure 3). The third example is the differentiation of lung BP into either AT1 or AT2^8^ (Figure 2b). The numbers of interactions in each TRN reconstruction step are shown in Supplementary Table S4 and the sources of remaining interactions are summarized in Supplementary Table S5. The predicted lineage specifiers are shown in Table 1.

Our method was able to predict experimentally validated lineage specifiers in the first two systems, which demonstrates the validity of the method. The method was further applied to less studied lung BP differentiation system, resulting in novel candidate lineage specifiers. As we used a general threshold logic rule for our Boolean simulation so as to keep our method applicable to any stem cell differentiation system, the robustness of our method to a different logic rule was assessed. To this end, the inhibitor dominant rule (where the presence of one inhibitor is enough to turn off the target gene regardless of the number of activators targeting the same gene) was employed, as this is one of the two most commonly used general logic rules (the other is the threshold rule). The predicted lineage specifiers based on this logic rule (Supplementary Table S6) were, albeit a few discrepancy, quite similar to the predictions based on the threshold rule, indicating that our method is not labile to at least these two most commonly used general logic rules.

Finally, each of our reconstructed cell subpopulation-specific TRNs exhibited unique topology in each differentiation event, giving a support to the previous statement that TRNs undergo significant rewiring during differentiation.^3^ Interestingly, we also attempted to reconstruct a single TRN whose attractors (both fixed point attractors and cyclic attractors) could satisfy three cell subpopulations in each differentiation event, however, our method was unable to find such a single TRN in all the five differentiation systems. The entire cell subpopulation-specific TRNs for the first two systems are shown in Supplementary Figure S2 and S3. The entire cell subpopulation-specific TRNs for the lung BP system were too large for meaningful visualization and are therefore not shown. However, all network files in sif formats are available in Supplementary Information 11–22. The most influential SCCs of the parental subpopulations are shown in Supplementary Figure S4. The predicted lineage specifiers in each differentiation event are discussed in detail in the following sections.

### ICM into PE and EPI

We predicted *Gata6* as a lineage specifier of PE, which has previously been shown to induce this differentiation.^36^ On the other hand, *Klf2* and *Nanog* were predicted for EPI cell-fate specification. *Klf2* has been strongly implicated in the establishment of EPI^13,14^ and *Nanog* is a well-known lineage specifier of this transition.^15^ Therefore, our prediction recapitulated known lineage specifiers in this system without predicting false positives.

### HSC into MPP or MEP

In this bifurcation event *Meis1* and *Gata1* are known to interact in MPP and the regulatory effect of *Meis1* on *Gata1* determines the specification of the erythropoietic cell lineage and inhibition of myelopoiesis.^37^ In addition, well-known myeloid-lineage specifiers *Cebpa*^16^ and *Spi1* (PU.1),^19^ and a known lymphoid-linage specifier *Gata3*^38^ were also predicted as lineage specifiers of MPP. The prediction of both of these lineage specifiers makes a biological sense since MPP has the potential for both lineages. In the HSC to MEP transition, not only *Gata1,* a well-known lineage specifier of the megakaryocyte–erythroid lineages,^17^ but also *Mbd2*, *Stat1* and *Trp53* were predicted. In accordance with this, there is strong experimental evidence of *Stat1* involvement in mediating the cell-fate decision between erythropoiesis and megakaryopoiesis.^39^ *Trp53* and *Mbd2* are not known to induce the MEP transition, and could be novel lineage specifiers. Apart from lineage specifiers, the most influential SCC of HSC correctly included active *Meis1* and *Gata2* (Supplementary Figure S4B), which are known to have an important role in the stability of HSCs.^6^

### MPP into CMP or CLP

In the MPP to CMP transition, not only *Stat1* but also myeloid-lineage specifiers *Cebpa*, *Gata2* and *Gfi1* were predicted.^18^ The fact that both erythroid-lineage and myeloid-lineage specifiers are active and involved in the stabilization core of CMP is another example (in addition to MPP mentioned above) of how two opposing lineage specifiers are co-expressed in the parental cell subpopulation and maintain its phenotype. In the MPP to CLP transition, a T-lineage gene, *Est1*,^40^ was predicted. Although the direct transition from the MPP to the CLP subpopulations was previously accepted,^41^ it is now known that LMPP is a more appropriate intermediate subpopulation that gives rise to GMP and CLP. However, the LMPP subpopulation was not profiled in the study from which we obtained the data set,^6^ and therefore we could not use this subpopulation. For this reason, the relatively small number of predicted lineage specifiers in this differentiation event could be because we did not consider lymphoid multipotent progenitor (LMPP), and used CLP instead.

### CMP into MEP or GMP

In the CMP to MEP transition, the predicted lineage specifier were *Gata1* and *Klf1*, the latter is known to be strongly involved in the establishment and maintenance of the erythroid lineage.^42,43^ In the CMP to GMP transition, the predicted lineage specifiers included well-known myeloid specifiers, such as *Cebpa, Gfi1* and *Spi1 (PU.1)*. Although *Nfat5* has been implicated in the regulation of lymphoid lineage, its role in GMP differentiation is unclear and this gene might be a novel lineage specifier.

### BP into AT1 and AT2

In the BP to AT1 transition, *Hes1* and *Mtf1* were predicted, whereas three genes (*Dbp*, *Pou6f1* and *Srf*) were predicted for the BP to AT2 transition. As expected, not so much is known about the lineage specification of BP into AT1 or AT2, however, *Hes1* is a Notch target in lung and has been implicated in mouse fetal lung development^20^ and *Pou6f1* has been shown to be associated with lung developmental pathway.^44^ In addition, D-site binding protein, *Dbp*, belongs to the bZIP protein family, and has been shown to bind to the promotor region of pulmonary surfactant *Sftpb*,^45^ which is formed by AT2. Finally, serum response factor, *Srf*, has been demonstrated to mediate TGF-beta induced differentiation of alveolar fibroblasts.^21^ Thus, many of the predicted lineage specifiers exhibit, to varying degrees, prior associations with lung development. Overexpression of these genes could induce respective lineage differentiation.

## Discussion

Stem cell differentiation is a complex event due partly to co-existence of different cell subpopulations within a heterogeneous population. These subpopulations exhibit different gene expression patterns and different propensities for lineage commitment. In the present study we have proposed a model of binary-fate stem cell differentiation, in which each stem cell subpopulation is in a stable state maintained by its specific TRN stability core, and cell differentiation involves changes in these stability cores between the parental and daughter cell subpopulations. Moreover, our model assumes that lineage specifiers for one daughter cell subpopulation should be differentially active in comparison to the other daughter cell subpopulation. This assumption excludes predictions of general prodifferentiation genes that are active in both lineages in comparison with the parental cell subpopulation. On the basis of this model, we have implemented a computational method for predicting subpopulation-specific lineage specifiers of binary-fate differentiation events.

To our knowledge, this is the first method that, without prior knowledge of potential candidate genes, systematically predicts cell lineage specifiers in stem cell subpopulations based on single-cell gene expression-based TRN reconstruction and a differential network analysis of the TRNs. Single-cell gene expression data are important for reconstructing TRNs, as TRN models generated from population-bulk expression data do not account for functional relationships between genes in each cell subpopulation.^22^ Furthermore,^3^ has observed significant TRN rewiring during differentiation. Hence, differential reconstruction of TRNs for different cell subpopulations appear to provide a biologically more realistic scenario than the reconstruction of a single TRN representing multiple cell subpopulations. The latter approach has been followed in previous single-cell-based studies for the prediction of cell lineage specifiers.^32,33^ Interestingly, our reconstruction of cell subpopulation-specific TRNs showed significant rewiring during differentiation and our computational approach could not find a single TRN whose attractors (both fixed point attractors and cyclic attractors) could satisfy the gene expression states of three different cell subpopulations.

The application of our method was able to predict well-known lineage specifiers (Table 1) in two well-studied examples,^4,6^ and predicted novel candidates of lineage specifiers in a less studied, lung BP differentiation system.^8^ These novel candidates have been previously shown to have some association with lung development, and could be experimentally validated in future. In addition, the lineage specifier predictions based on the inhibitor dominant rule (Supplementary Table S6) were, albeit a few difference, more or less the same as the predictions based on the threshold rule, indicating that our method is not labile to these two most commonly used general logic rules. Finally, given that each cell subpopulation consists of single cells with varying expression states, a possible future work would be to represent these substates within a subpopulation with a non-fixed point attractor (e.g. cyclic attractor). Such an approach could enable us to understand which single-cell states are more prone to differentiation into a certain lineage. Furthermore, the development of an *in silico* method for simulating network rewiring could provide mechanistic insights into the molecular dynamics during differentiation.

As the production of single-cell gene expression data is rapidly increasing, we believe that approaches like the one presented here would be useful for the identification of lineage specifiers and cell subpopulation-specific TRN stability cores. Such understanding should help design experimental cell differentiation protocols with higher efficiency and fidelity, and aid in regenerative medicine.

## Figures and Tables

**Table 1:** Predicted lineage specifiers in each binary-fate differentiation step

*Data set*	*Parental cell population*	*Daughter Cell Population*	*Predicted lineage specifiers*
Guo *et al.*^4^	ICM	PE	***Gata6***
		EPI	***Klf2**, **Nanog***
Guo *et al.*^6^	HSC	MPP	***Cebpa**, Etv6, **Gata3**, **Meis1**, **Spi1***
		MEP	***Gata1**, Mbd2, Stat1, Trp53*
	MPP	CMP	*Cebpa, Gata2, Gfi1, Stat1*
		CLP	*Ets1*
	CMP	MEP	***Gata1, Klf1***
		GMP	*Ahr, **Cebpa**, **Gfi1**, Nfat5, **Spi1**, Runx1*
Treutlein *et al.*^8^	BP	AT1	*Hes1, Mtf1*
		AT2	*Dbp, Pou6f1, Srf*

Abbreviations: BP, bipotential progenitor; CLP, common lymphoid progenitor; CMP, common myeloid progenitor; EPI, epiblast; GMP, granulocyte–macrophage progenitor; HSC, hematopoietic stem cell; ICM, inner cell mass; MEP, megakaryocyte–erythroid progenitor; MPP, multipotent progenitor; PE, primitive endoderm; SCC, strongly connected component; TRN, transcriptional regulatory network.

Each binary-fate differentiation step is indicated with a combination of parental cell subpopulation and daughter cell subpopulation. Genes in bold are known lineage specifiers for that cell subpopulation.
