# Supplementary material for: A differential network analysis approach for lineage specifier prediction in stem cell subpopulations
Source: NPJ Syst Biol Appl. 2015 Nov 12;1:15012–. doi: 10.1038/npjsba.2015.12 (PMC5516870; doi:10.1038/npjsba.2015.12)
Supplement: Supplementary Information [file npjsba201512-s7.pdf]

Supplementary Materials

Supplementary tables

- **Table S1**: Booleanized gene expression for the Guo et al. (2010) data set**.**
- **Table S2**: Booleanized gene expression for the Guo et al. (2013) data set**.**
- **Table S3**: Booleanized gene expression for the Treutlein et al. (2014) data set**.**
- **Table S4**: Numbers of interactions (network edges) in each TRN reconstruction step**.**
- **Table S5**: Sources of TRN interactions. The literature-based interactions are supplemented with respective PubMed IDs and predicted interactions (predicted from both coexpression and TF-DNA binding motif prediction) are labelled “predicted”.
- **Table S6**: Predicted lineage specifiers based on inhibitor dominant logic rule.

Supplementary codes and data files for GRN contextualization and most influential SCC finding

1. Programme files

- **Supplementary Information 1** (doContextualization.m): MATLAB programme for GRN contextualization
- **Supplementary Information 2** (myCircuits.m): MATLAB programme called from within doContextualization.m
- **Supplementary Information 3** (myOptimFun.m): MATLAB programme called from within doContextualization.m
- **Supplementary Information 4** (myBooleanSimulation2.m): MATLAB programme called from within doContextualization.m
- **Supplementary Information 5** (process_contextualized_network.R): R script for processing contextualization output for most influential SCC finding
- **Supplementary Information 6** (findSCCs.m): MATLAB programme for finding SCCs
- **Supplementary Information 7** (findCircuits2.m): MATLAB programme called from within findSCCs.m
- **Supplementary Information 8** (adjacencyToSif.R): R script for converting all SCC adjacency matrix files into .sif files and make a summary file of all SCCs
- **Supplementary Information 9** (computeInfluentialSCCs.R): R script for computing the influence of each SCC
- **Supplementary Information 10** (findLineageSpecifiers.R): R script for finding candidate linage specifiers

2. Data files

- **Supplementary Information 11 - 22** (*_raw_network.sif): raw networks for the used cell subpopulations in sif format
- **Supplementary Information 23 - 34** (adjacency_matrix_*_raw_network.txt): adjacency matrices of raw networks for the used cell subpopulations.
- **Supplementary Information 35 - 46** (Booleanized_*.txt): Booleanized gene expression for the used cell subpopulations.
- **Supplementary Information 47 - 56** (up_*-*.csv): log2 fold-change expression higher in one daughter subpopulation in comparison to the other daughter subpopulation

Programme Usage (ICM, PE and EPI differentiation as a toy example)

1. Download MatlabBGL and pbn-matlab-toolbox packages and "graphkshortestpaths.m" programme. These are freely available on the web.
2. Prepare necessary files (adjacency matrix for raw network, Booleanized expression file) for each cell subpopulation
3. Change the input file names for "doContextualization.m" accordingly.
4. *Run "doContextualization.m"
5. ‡Run "process_contextualized_network.R" (*e.g. ./process_contextualized_network.R adjacency_matrix_ICM_raw_network.txt GA_bestSolution.txt no*)
6. Make a "SCCs" directory for the "findSCCs.m" programme output
7. Specify the raw network file, Booleanized expression file, "SCCs" directory name, "adjacency_matrix_best_GA_bestSolution.txt" file, "nonzeroIndices_GA_bestSolution.txt" file and "adjacencyVectors_GA_bestSolution.txt" file in the "findSCCs.m" programme
8. *†Run "findSCCs.m"
9. Run "adjacencyToSif.R" (e.g., *./adjacencyToSif.R SCCs*)
10. Run "computeInfluentialSCCs.R" (*e.g. ./computeInfluentialSCCs.R summary_SCCs.txt adjacency_matrix_best_GA_bestSolution.txt Booleanized_ICM.txt SCCs/*).
11. Complete 1-9 for one binary-fate differentiation event (i.e., three subpopulations)
12. Run "findLineageSpecifiers.R" to find predicted lineage specifiers for one daughter cell subpopulation from the parental one. (*e.g. ./findLineageSpecifiers.R F_summary_SCC_EPI.txt.Rdata Booleanized_EPI.txt F_summary_SCC_PE.txt.Rdata Booleanized_PE.txt F_summary_SCC_ICM.txt.Rdata Booleanized_ICM.txt up_EPI-PE.csv 5*). The last input argument is the top ranks of the most influencial SCCs that will be taken. 5 was used for the first two systems and 10 was used for the lung system in order to take into account the different network sizes. The choice of this parameter is up to the user and this gives some flexibility in terms of cutoff stringency.

*These programmes were run on high-performance cluster in the original study.
‡In general, the topology of a contextualized TRN can differ slightly in each run due to the heuristic nature of the genetic algorithm used in this method. However, the genes within SCCs almost always remain the same and we obtained the same lineage specifier predictions in our trials.
†This programme is not optimized for efficiency. For example, the number of first shortest paths from each node is set to 5000 throughout this study to cover the enough graph space, however, this could be smaller for small networks.

It is important to make sure that the attractor mismatch is almost 0 after running the doContextualization.m programme. In the example cases, usually 1000 populations and 100 generations are enough.

Supplementary Figures

Figure S1: Sources of information for building TRNs. Venn diagram showing the set of interactions used in TRN. As described in Materials and Methods, we combined TF-TF interaction information from literature (extracted from MetaCore™) (green), TF-DNA binding site predictions (magenta) and single cell co-expression information (red). We intersected the interactions coming from these three sources of information and took the union of the intersections between co-expression and TF-DNA predictions with co-expression and literature information (in red).

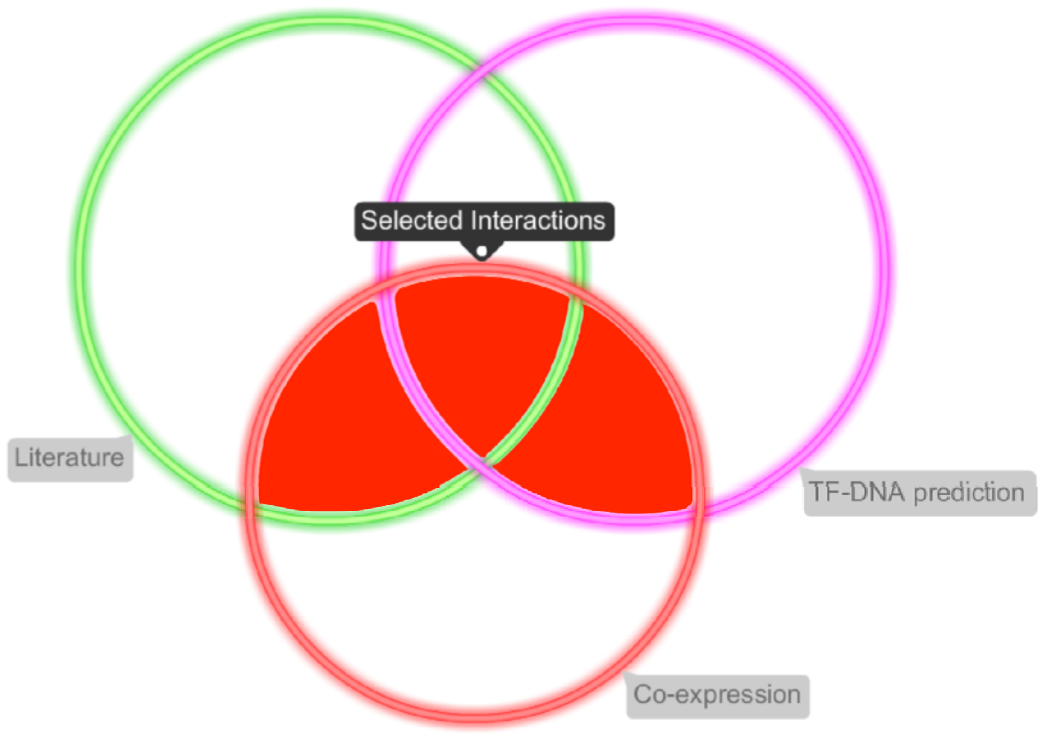

Figure S2: Transcriptional regulatory networks of stem cell subpopulations in early embryonic development. The TRNs inferred by using single cell data-based gene co-expression and a combination of literature information for TF-TF interactions and TF-binding site predictions are shown for the three cell-types analyzed in this study . In the figure each panel correspond to: A), ICM B) PE, C) EPI. Red nodes indicate differentially active genes and blue nodes indicate non-differentially active genes.

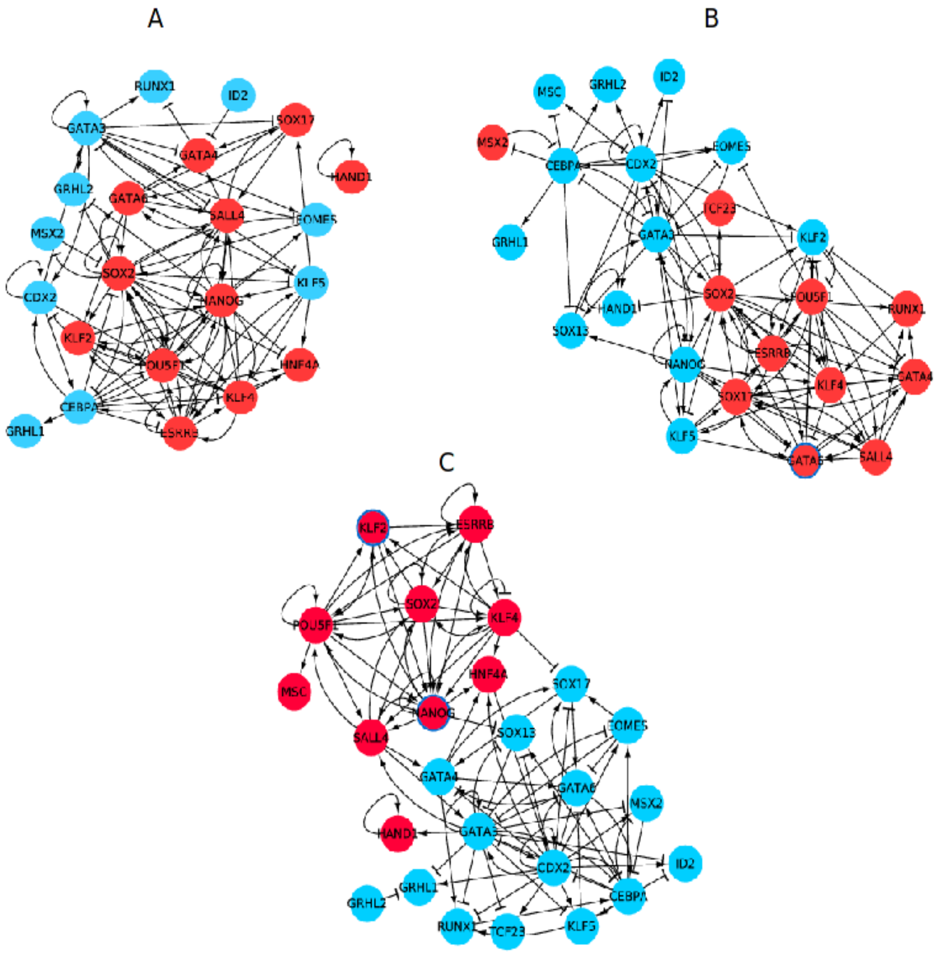

Figure S3: Transcriptional regulatory networks of stem cell populations. TRNs inferred by using single cell data-based gene co-expression and a combination of literature information for TF-TF interactions and TF-binding site predictions are shown for the six stem cell subpopulations analyzed in this study. In the figure each panel correspond to: A), HSC B) MPP, C) MEP, D) CMP, E) CLP and F) GMP. Red nodes indicate differentially active genes and blue nodes indicate non-differentially active genes.

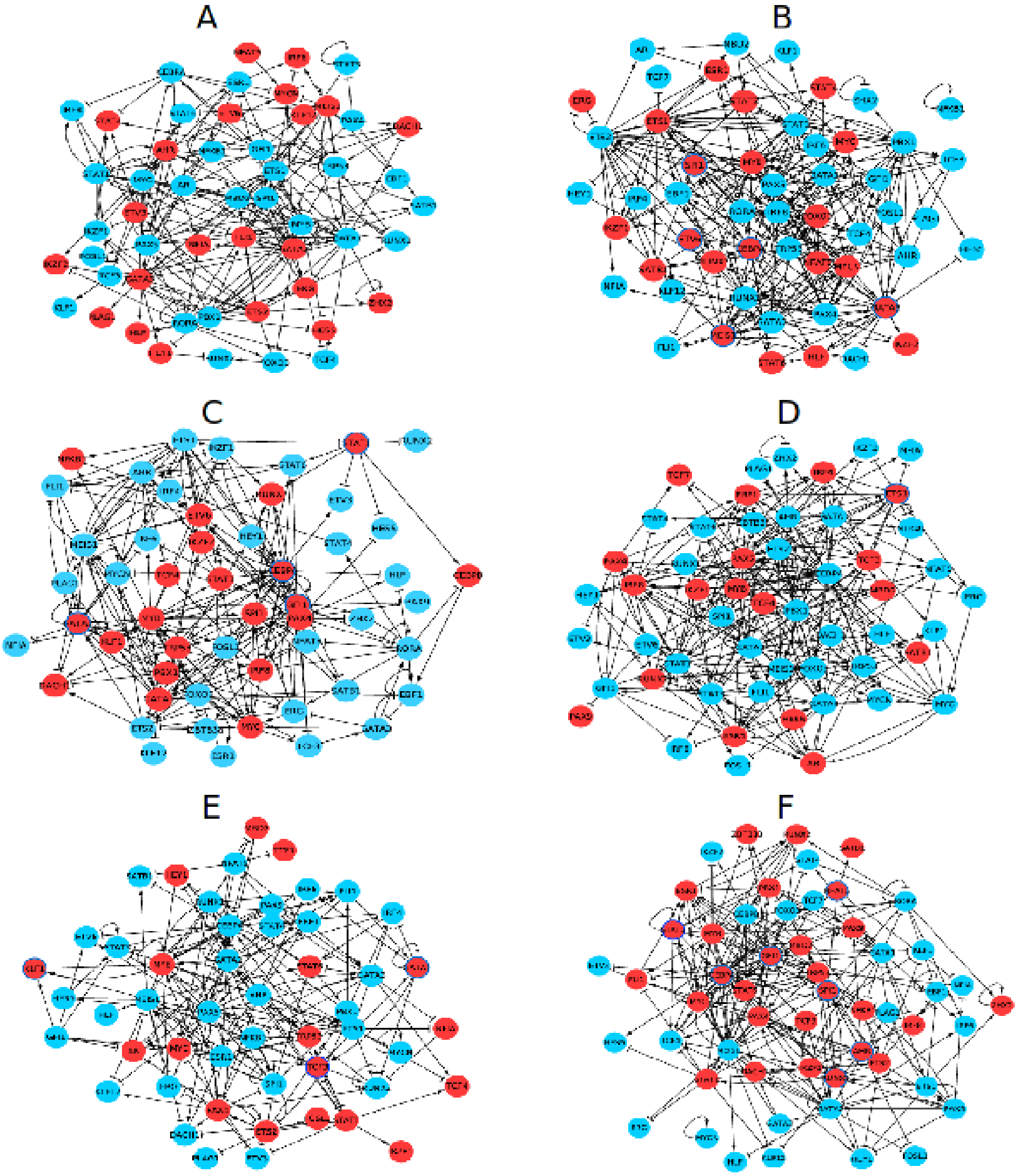
A
B
C
D
E
F

Figure S4: The most influential strongly connected component stabilizing the parental cell subpopulations. We show the most influential SCCs in the TRN of A) ICM, B) HSC and C) BP, which are related to the stabilization of respective phenotypes. Red nodes indicate differentially

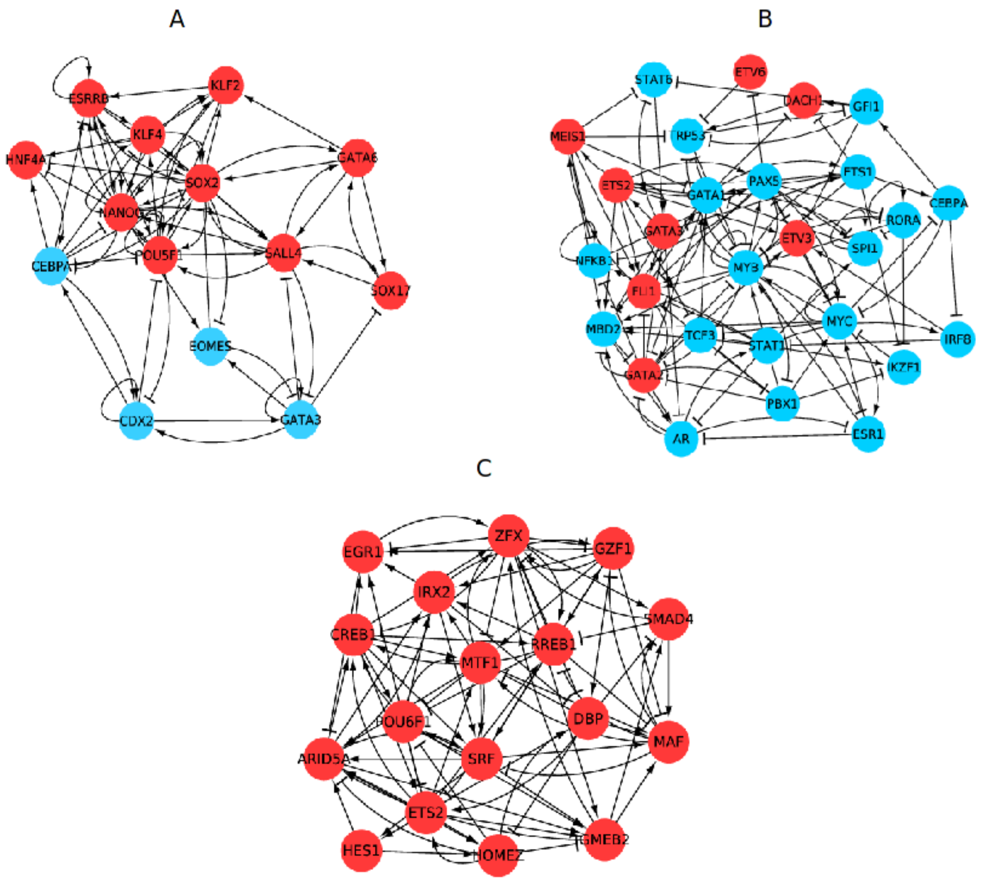

active genes and blue nodes indicate non-differentially active genes.
